# Building Partnership to Improve Migrants’ Access to Healthcare in Mumbai

**DOI:** 10.3389/fpubh.2015.00255

**Published:** 2015-11-16

**Authors:** Nilesh Chandrakant Gawde, Muthusamy Sivakami, Bontha V. Babu

**Affiliations:** ^1^School of Health Systems Studies, Tata Institute of Social Sciences, Mumbai, India; ^2^Division of Health Systems Research, Indian Council of Medical Research, New Delhi, India

**Keywords:** migration, healthcare, process evaluation, intervention, community participation

## Abstract

**Objectives:**

An intervention to improve migrants’ access to healthcare was piloted in Mumbai with purpose of informing health policy and planning. This paper aims to describe the process of building partnership for improving migrants’ access to healthcare of the pilot intervention, including the role played by different stakeholders and the contextual factors affecting the intervention.

**Methods:**

The process evaluation was based on Baranowski and Stables’ framework. Observations in community and conversations with stakeholders as recorded in daily diaries, minutes of pre-intervention workshops, and stakeholder meetings served as data sources. Data were coded using the framework and descriptive summaries of evaluation components were prepared.

**Results:**

Recruitment of stakeholders was easier than sustaining their interest. Community representatives led the intervention assisted by government officials. They planned community-level interventions to improve access to healthcare that involved predominantly information, education, and communication activities for which pre-existing formal and informal social networks and community events were used. Although the intervention reached migrants living with families, single male migrants neither participated nor did the intervention reach them consistently. Contextual factors such as culture differences between migrants and native population and illegality in the nature of the settlement, resulting in the exclusion from services, were the barriers.

**Conclusion:**

Inclusive multi-stakeholder partnership, including migrants themselves and using both formal and informal networks in community is a feasible strategy for health education and has potential to improve the migrants’ access to healthcare. However, there are challenges to the partnership process and new strategies to overcome these challenges need to be tested such as peer-led models for involvement of single male migrants. For sustaining such efforts and mainstreaming migrants, addressing contextual factors and having formal mechanisms for their inclusion are equally important.

## Introduction

According to estimates, 763 million people were internal migrants, i.e., living within national borders but outside their region of birth in 2005 accounting for more than 10% of global population (1). India has the largest share of internal migrants with more than 300 million reported in the census of 2001 (2). However, lack of explicit policy framework for poor migrants excludes them from access to legal rights, social security, and public services, including basic amenities such as water and sanitation (3, 4). There is evidence of exclusion of poor migrants from healthcare in urban India (5–8). Efforts to provide healthcare to migrants have been largely in the context of infectious diseases, especially HIV, tuberculosis, and malaria where migrants are often viewed as infection source resulting in stigmatization (9–12). Such disease-specific approaches aimed at reducing the burden of a specific disease may not address disparities in health. From the rights perspective, it is imperative to have mechanisms that improve overall health of migrants, reduce inequities, and achieve social inclusion. Access to healthcare is one such comprehensive strategy (13).

Migrant communities tend to underutilize healthcare services because they are alien to the urban system and are away from the traditional and government healthcare systems available in their place of origin. Such lack of access to healthcare services in the public sector often drives poor migrants to access services from the private healthcare settings that result in high out-of-pocket expenditures with consequent impoverishment. Access to private healthcare providers is not easy either. Although urban areas have far greater number of private healthcare providers, they are functionally inaccessible to majority of the urban poor, especially the migrants. Cost, timings, distance, attitude of health providers, and other factors put the secondary care and private sector facilities out of reach for most urban poor (14). The latter, especially migrants, often settle for cheaper but unsafe and poor quality services provided by unqualified providers. Studies across countries have documented resultant poor access to healthcare for migrants (5, 15, 16). In the light of issues, including migrants’ poverty, lack of health insurance, high out-of-pocket expenditure, uncertainty of quality services in unregulated private sector, and role of government to provide affordable healthcare to poor and marginalized, it is crucial to identify ways to improve migrants’ access to healthcare from the public sector.

National Health Mission (NHM) of India in which National Urban Health Mission (NUHM) is a part, envisages making essential primary healthcare services available to urban poor, especially migrants, and reducing their out-of-pocket expenditure. However, currently there are both demand and supply side challenges in delivery of healthcare to poor migrants (17). Keeping this in mind, a pilot intervention was planned to improve access to healthcare among poor recent migrants in Mumbai, India. Learning from this intervention was expected to help NUHM in devising appropriate strategies to meet its aim of reducing urban disparities in health.

Present intervention was based on two core strategies: community participation and partnership. Community participation is one of the foundational principles of primary health care with evidence of improvement in health services’ utilization (18, 19). Also, ethical and rights perspective lays greater emphasis on community participation that can generate demand for healthcare. Health system responsiveness is equally important in order to provide quality services that are accessible to poor migrants. For this, partnership between various stakeholders, including suppliers, users, and facilitators was felt necessary. Hence, an inclusive partnership strategy, including all the stakeholders, was conceptualized and the research team played as facilitators in the entire intervention process.

Interventions in the area of migrants’ access to health are sparse and would benefit from evaluations of such attempts to inform research and policy. It was, therefore, very important to understand the process of building partnership, who were the key drivers of the intervention, what the contribution of each stakeholder was, what was the extent of participation by community, how did the intervention strategies emerge, whether the intervention was implemented as planned by stakeholders, and what the barriers to intervention were. Process evaluation can help address such questions and possibly explain reasons behind success or failure of an intervention (20–23). The aim of this paper on process evaluation is to document successes and challenges of the intervention with migrants (community) and other stakeholders as partners in designing, planning, and implementing intervention. To the best of our knowledge, there is no single paper published on the process of intervention efforts to improve access to healthcare for internal migrants in India that identifies positive aspects that may guide policy formulation and implementation of future interventions under NUHM.

## Materials and Methods

### Study Settings

Mumbai metropolitan region is one of the largest urban agglomerations with nearly 1.8 billion residents (24). Out of all cities in India, total number of in-migrants during 1991–2001 was the largest for Mumbai (2). Livelihood is principal reason for this internal migration from backward states of India with less opportunity of employment and poor wage rates. Displacement due to conflict or developmental projects does not contribute significantly. The city was de-industrialized in 1980s and 1990s and a majority of the jobs are in the unorganized sector (25). Its vast unorganized sector is characterized by smaller manufacturing units (often not registered) employing few individuals, no guarantee of job for employees, hardly any division of labor, and lack of social security, and health insurance. Many such units operate within slum areas and some of them also work as work-cum-residential units. Poor migrants working in these settings have lower wages and suffer deprivations in housing, basic amenities, including water supply, sanitation and are often excluded from social security programs.

### Healthcare in Mumbai

Municipal Corporation of Greater Mumbai (MCGM), the local administrative body, is responsible for provision of health services to its residents. It provides primary health care through Urban Health Posts each of which covers a defined geographic area and provides both preventive and curative care. Curative care is provided by dispensaries (primary level), peripheral hospitals (secondary level), and medical college hospitals (tertiary level). There are many non-governmental not-for-profit organizations (referred to as NGOs since this is term used in India rather than NPOs) that provide a variety of social and health services to slum dwellers in many slum areas. NGOs depend predominantly on funding agencies for carrying out interventions. Other healthcare providers (for-profit sector) include non-qualified and qualified practitioners and hospitals.

### Selection of Community Clusters

Most of the poor migrants live in slums or in make-shift residential structures at construction sites. The chosen ward was identified for conducting intervention as it had both single male migrants and migrant families, had lowest Human Development Index within Mumbai and its proximity to the research institute. Since this was a pilot intervention, it was planned for three community clusters only. Criteria for selection included sufficient size of the cluster (500–1000 households), a considerable proportion of recent migrants (more than 20% of population in it having migrated in the previous 10 years), its existence for at least one and half years and its not having mobile population. Construction sites were not considered for intervention as construction workers move from the site after completion of the project (mobile population), which would lead to interruption in intervention. Five clusters fulfilling these criteria were identified, out of which three were chosen randomly.

### Intervention

Aim of the intervention was to improve utilization of government health services among migrants, especially recent ones who had migrated during the previous 10 years. Inclusive partnership strategy was employed involving diverse and inclusive representation of all stakeholders/participants. The research team visited intervention clusters frequently from March to May 2013 to identify stakeholders, including community members (migrants in selected clusters) and functionaries from NGOs with projects in health domain, community-based organizations (CBOs), and MCGM that would be interested in designing and implementing the interventions. Table 1 summarizes major activities that began with identifying stakeholders for selected community clusters. The research team (authors, research associate, and two research assistants) did not design or implement the intervention, but it facilitated meetings and interventions of the core groups.

**Table 1:** **Gantt chart of intervention to improve migrants’ access to healthcare, Mumbai**.

Activity	March 2013	April 2013	May 2013	June 2013	July 2013	August 2013	September 2013	October 2013	November 2013	December 2013	January 2014	February 2014	March 2014
Identifying stakeholders													
Pre-intervention workshops													
Formation of core group in each cluster													
Interventions in community													
Process evaluation													

*Gray shades indicate the time period during which the activity took place*.

#### Community Participation

The term “community participation” has been understood differently by different researchers. McLeroy and others (26) point out that researchers have used the term community-based interventions in the past to reflect settings in which the study takes place, community being the target of intervention, a resource with high degree of community ownership and partnership, and the final one includes community as agent (26). In the first two types, community participation is much more passive, whereas in the subsequent two types, it is more active. Community as “agent” of change has been rarely used in public health interventions (26). Recognizing the importance of community participation, it was planned that intervention and its activities would emerge from the needs of migrants themselves and be driven by them and other stakeholders in the community instead of researchers. In the present intervention, the term “community” refers to people living in the geographical area of the three selected intervention clusters. The community was a mix of settled and recent migrants.

#### Inclusive Partnership

After identifying the stakeholders, the task was to bring them together, forge partnership, and design intervention for which the research team played the role of a facilitator. For this, three pre-intervention workshops were conducted. Representatives of all stakeholders participated in them. During the pre-intervention workshops, results from formative phase of the study on healthcare access to migrants in Mumbai were presented to stakeholders in order to initiate discussion among them regarding the possible ways to intervene. Discussions among stakeholders identified most common reasons for not accessing government health facilities that included lack of knowledge regarding location, timing, and services provided at them. By the end of third workshop, broader concept of intervention evolved with health education and promotion as its central focus.

The stakeholders formulated objectives and listed interventions to be conducted. The intervention designers felt that the intervention cannot be structured and delivered to the three selected communities in an identical manner. The architects of intervention planned to have core groups, one for each community with the task of preparing cluster-specific action plan and monitoring the progress of intervention. The core group for each community included five to eight representatives from the migrant community, medical officer/s of urban health posts catering to that community cluster, and NGOs with health project in and around it. It was proposed that every core group would meet once a month. During the core group meetings, stakeholders discussed interventions that emerged during pre-intervention workshops, refined the list of activities needed, identified resources required, assigned tasks among themselves, reviewed progress achieved, identified barriers, and implemented changes in the delivery of intervention. The interventions agreed upon were implemented from July 2013 to March 2014. Details of proposed interventions are provided in Table 2.

**Table 2:** **Intervention to improve migrants’ access to government healthcare services: summary of intervention components as proposed by stakeholders, Mumbai**.

Intervention components	Need of intervention	Preparatory steps	Means of executing intervention
IEC regarding time and location of specific health services provided by MCGM	Migrants were not aware about health services (types of services, their timings, and locations) offered by government and about their time and location	Schedule of health services and their location was prepared for each of the three clusters by medical officers of urban health post. The schedule was translated in Hindi. Materials included pamphlets, posters, and flip charts	(1) Display at prominent locations within community, (2) Display at community festive events, (3) Distribution through community self-help groups and informal networks of core group members
IEC regarding common health issues relevant to slums of Mumbai	Many migrants were not aware of the health risk posed by their environment (for example, unsafe water, mosquitoes, etc.), were not aware of preventive measures, and did not know when to seek healthcare and where	IEC cell of MCGM prepares and tests health education material for specific health condition. The IEC material in Hindi and Marathi covering prevention and treatment of common health conditions, maternal, and child healthcare was reproduced. Materials included pamphlets, posters, and flip charts	(1) Display at prominent locations within community, (2) Display at community festive events, (3) Distribution through community self-help groups and informal networks of core group members
Exposure visits to government health facilities	Migrants have not seen facilities available at government health centers and are not aware about basic administrative steps while seeking care	Medical officers to chalk out plan for exposure visit to primary and secondary level government health facilities catering to the intervention clusters	(1) Exposure visit of migrants’ representatives to government health facilities
Workshop on communication skills for staff of maternity home	Community members pointed unsatisfactory behavior of staff that pushed migrants to either out-of-pocket expenses in private sector or unsafe home deliveries	IEC cell of MCGM designed workshop on communication skills for staff	(1) Workshop conducted by IEC cell
Service provision in community (immunization camp, direct observation of TB treatment)	Migrants, especially women, face barriers in reaching health facility (not aware of city, nobody to look after another child at home, timings of water supply erratic, and clash with facility timings)	Identifying a community volunteer for provision of direct observation of TB treatment, planning immunization camp in community at location, and time convenient to migrants	(1) Scheduling immunization camp at fixed location, time, and date in community and (2) Training of community volunteers for direct observation of TB treatment
Strengthen human resources of community	Sustaining intervention would need a number of peer leaders among migrants with requisite skills	Identifying representative for every 10–20 households or for each industrial unit	(1) Capacity building of community volunteers

*MCGM, Municipal Corporation of Greater Mumbai; IEC, Information, Education and Communication; TB, tuberculosis*.

### Process Evaluation

As mentioned earlier, objectives of this process evaluation were to describe what role was played by stakeholders in design and implementation of intervention, how the intervention was implemented, which contextual factors affected the intervention, and what were the barriers. Relevant process evaluation frameworks were searched for in the literature. Baranowski and Stables (27) developed process evaluation components and applied them in diverse community health promotion projects (27). Although there are other frameworks (21, 28) on the process evaluation, these models were more suitable for interventions with structured interventions. However, the present intervention was designed and planned by stakeholders and interventions evolved over a period of time, researchers having no role in determining activities to be undertaken. It was important to assess various stakeholders’ role in this community-based health promotion and health education intervention. Hence, the process evaluation components proposed by Baranowski and Stables (27) were found to be more appropriate for the present intervention as it had components that could assess recruitment, maintenance, and role played by all the stakeholders.

For assessing the stakeholders’ participation in design and implementation of intervention, three process evaluation components were identified. The process evaluation component, recruitment, and maintenance aimed to examine the process by which various stakeholders got interested in the intervention, ease or difficulty in joining the intervention and whether the interest was maintained during the intervention period. Component on stakeholder analysis was added that reflected the role played (supporter/blocker/bystander) by each stakeholder and who were the drivers of intervention. For understanding implementation of intervention and barriers faced during the same, six components, including resource, implementation, reach, barriers, exposure, and contamination were selected. Context component helped in understanding contextual factors affecting intervention. Two components of initial and continued use were not considered for process evaluation because utilization of healthcare applied to only those who fell sick. Selected components were operationally defined for this study (see Table 3).

**Table 3:** **Components of process evaluation of intervention to improve healthcare access to migrants**.

Component	Description
Recruitment	Attracting stakeholders, including representatives of migrants and host communities, non-governmental organizations, health personnel from municipal corporation, religious leaders in community, and local politicians for developing and implementing intervention
Maintenance	Keeping stakeholders involved in the program design and implementation
Stakeholders’ role	Role played by stakeholders in design and implementation of intervention
Context	Aspects of the environment of an intervention
Resources	Human, financial, and material resources contributed by which are necessary to attain project goals
Implementation	The extent to which the program is implemented as designed by stakeholders
Reach	The extent to which the program contacts or is received by the targeted group (migrants)
Barriers	Problems encountered in implementing various intervention components
Exposure	The extent to which participants view or read the materials that reached them
Contamination	The extent to which participants receive interventions from outside the program and the extent to which the control group receives the treatment

*Modified from Baranowski and Stables (27)*.

### Data Sources

Qualitative approach was used for process evaluation. The research team visited all the three community clusters during intervention and attended core group meetings in the community. It filled daily diaries with observations in the field and conversations with stakeholders related to intervention as well as other contextual issues. Minutes of workshops and core group meetings were also recorded. All of this served as data source for process evaluation. Details of data sources used for process evaluation components of the study have been presented in Table 4. The process evaluation was conducted by the research team, but it was not involved in planning or implementation of intervention as stated already.

**Table 4:** **Source of data for process evaluation components**.

Component	Conversations with stakeholders before dissemination field visits	Minutes of dissemination workshop	Minutes of core group meetings	Observations in community	Conversations with stakeholders during intervention
Recruitment	√	√			
Maintenance			√	√	√
Stakeholders’ role	√	√	√	√	√
Context			√	√	√
Resources			√	√	√
Implementation				√	√
Reach				√	√
Barriers			√	√	√
Exposure				√	√
Contamination				√	√

### Analytic Approach

All the qualitative data (minutes of meetings and workshops, observations, and conversations) were transcribed and typed. ATLAS-Ti (version 6.2; ATLAS.ti GmbH, Berlin, Germany) was used to code data. Transcripts were read and re-read and codes were identified using Baranowski and Stables’s framework. Broader themes were identified by collapsing of codes and descriptive summaries related to each process component were written. Reflection notes of first two authors were used to verify conclusions drawn from the data. Stakeholders were mapped by their level of interest in the issue, influence on intervention, and extent of participation in intervention. They were classified as drivers, supporters, blockers, bystanders, and abstainers with the help of classification used by Namazzi et al. 2013 (29).

### Ethical Considerations

The study was conducted in accordance with the principles from the Declaration of Helsinki. It was approved by the Institutional Review Board of the Institute of first two authors. All stakeholders were assured that their responses would be treated anonymously. In this paper, only pseudonyms have been used to protect the identity of studied participants.

## Results

### Recruitment of Stakeholders

Identifying and recruiting stakeholders was relatively a smooth process. The research institute of first two authors has longstanding partnership with NGOs working in the area and MCGM for field action projects, research projects and advocacy interventions. However, the previous collaborations were not related to migrants or healthcare access to people. Upon receiving invitation letters, stakeholders (from NGO and MCGM) readily agreed to participate and confirmed their participation in a workshop for conceptualizing intervention. The participants from MCGM and NGO were mostly graduates or with professional training in health interventions.

Stakeholders also knew one another well. NGOs had partnered in recent past with governmental machinery for health-related interventions. Government health officials and NGOs engaged services of community volunteers as link between program planners and community. In addition, developmental NGOs had facilitated creation of women’s self-help groups and had built capacity of volunteers from the community in delivering healthcare.

Conversations with the community and health workers of MCGM and NGOs helped identify community members with good social networks and having interest in devoting time for community-level activities. Some of them were members of formal social groups (for example, *Ms.Farhana* (name changed) from cluster 1 was a member of women’s self-help group) and some others had informal social networks as reported by *Mr. Akhtar* (name changed) from cluster 3. All such members from the community were literate but almost none had completed secondary school level. They had no training in public health but mostly had experience of working in developmental projects as volunteers.

These community volunteers shared the idea in their formal and informal networks and such people and/or groups were met by the research team during subsequent visits. Cluster 3 needed only three visits as the research team could meet interested community members with larger informal networks (for example, *Mr. Gavali* reported 60 people in his network) earlier, whereas other two communities needed seven field visits to identify the interested members. These volunteers became members of the core groups.

However, most of the interested members were settled migrants (who migrated to Mumbai 20–25 years ago). Recent migrants (who migrated within the past 10 years) neither volunteered themselves nor were they identified by the community as its representatives. Out of 29 members who participated in pre-intervention workshops, only two had migrated within previous 10 years, 25 were settled migrants, and Mumbai was birthplace for the remaining two. Recent migrants did not have large networks, had limited sphere of influence and had not participated in community-level activities prior to this intervention.

Single male migrants stayed away from design and implementation, none of them participated in pre-intervention workshops and volunteered to be a core group member. This group was isolated in terms of social interactions with the rest of the community and the latter could not rope them in. The research team explored their interest for participation. One migrant working in *zari* unit said, “*I am not interested as I would not like to lose my pay*.” At another garment unit, a migrant said, “*Five of us stay here but we are not interested in coming for a meeting on health; none of us is sick*.”

### Maintenance

Although core groups were expected to meet each month, they met once in 2 months and the total number of meetings was five, four, and two for first, second, and third clusters, respectively. The third cluster had fewer meetings due to external reasons. Anti-encroachment department of the MCGM demolished the non-notified residential structures during the intervention. This led to displacement of the residents and hence fewer meetings. Interventions suffered interruptions and were mostly stopped over period of 3 months (October–December 2013). The other two clusters have experienced similar demolitions in the past but were not affected during the intervention period of this study.

Response during the first core group meetings of the three clusters was encouraging. Government health officials attended most meetings at community level. Their participation persisted throughout the intervention. However, the research team had to facilitate meetings and remind health officials about their plans periodically. While the NGO representatives attended nearly all the core group meetings and facilitated discussion, they did not take part in implementing intervention.

Although the core group decided to invite other community members who were not its part to participate in the intervention, their participation did not continue. Only those members who were associated with CBOs continued to support the core group members and assisted them in implementation. Minimal participation by single male migrants did not continue.

### Stakeholders’ Role

Stakeholders had varied levels of engagement with intervention. They differed in the level of interest in intervention, power/influence, type of participation, and supporting intervention (Table 5).

**Table 5:** **Stakeholders’ analysis: stakeholders’ interest, power/influence, and role in intervention**.

Type of stakeholders	Interest in issue	Level of power/influence	Type of participation	Classification of stakeholders
Recent migrants living with families	Migrants in need of healthcare, desire improved access to healthcare	Moderate influence	Beneficiaries	Moderate level supporter
Settled migrants living with families	Same as above	High influence but limited skills and resources	Beneficiaries and participated in implementing intervention	Moderate level supporter
Single male migrants	Same as above	Little influence	Beneficiaries	Low level supporter
Migrants who were members of core group	As above and in addition willingness to work on voluntary basis toward welfare of community	High influence, basic skills but limited resources	Decision makers, beneficiaries, and providers of intervention	High-level supporter
Government health officials and workers	Responsibility for provision of health services in community	High influence but limited human resource	Decision makers and service providers	High-level supporter
Representatives of NGOs	NGOs provide healthcare, link community with government health services, and aim at empowering community	Little influence	Facilitating decision making	Bystanders

*NGO, non-governmental (not-for-profit) organization; CBO, community-based organization; the term includes women’s groups, youth clubs (both registered and unregistered)*.

#### Recent Migrants Living with Their Families

They were more vulnerable than settled migrants as often they did not know about location, timings, and types of government health services available in their areas. They had limited interactions with the community and very few of them were members of CBOs. Settled migrants shared the social networks within neighborhood developed over years by their stay in Mumbai and so participated in community affairs. Among recent migrants, those living with families could be approached by the existing women’s groups. They conveyed to core group members their lack of awareness regarding location, timings, and health services of MCGM and difficulties faced while accessing them. Although this helped design appropriate interventions, the recent migrants themselves did not take active role in planning or executing the same. These families remained only beneficiaries and received Information, Education, and Communication (IEC) materials and messages through core group members and women’s groups.

#### Settled Migrants Living with Their Families

Settled migrants knew more about the availability of healthcare services in both public and private sectors. Their social networks had developed over the years and many were members of youth clubs and women’s self-help groups, and participated in community-level activities including festivals. These families were particularly aware of their rights over houses, water, sanitation, and health. During meetings, they communicated about the barriers in accessing government healthcare services and some of them also participated in planning and implementation. For example, some youth clubs displayed IEC material at festive occasions and women’s groups used flip charts and pamphlets to disseminate the IEC messages. But they had limited skills and resources; the major resource that they could contribute was their time.

#### Single Male Migrants

They were employed in many small-scale industrial units, often informal in nature. Some of them worked in units away from their residence and were available only on their weekly offs (mostly Sundays). Those working within the slum clusters also did not spare time during the day due to fear of losing wages. Timings suitable to them (late evenings) were not suitable to other stakeholders. Their lack of time prevented them from informing core group members about their health needs. Most of them were non-committal about devoting time either for planning or implementation. The group had little influence on the intervention.

#### Migrants Who were Members of Core Group

They were the “torchbearers” of intervention. They used meetings of housing colonies, youth clubs, and women’s self-help groups to list the health needs of the community. They had fair understanding of the community needs through their life experiences that helped them in articulating their needs during their interactions with the community as well as other stakeholders. They followed up with government health officials regarding activities to be undertaken. They had notable influence in design and planning intervention. This group carried out intervention activities with the help of women’s self-help groups and youth clubs. It contributed its time, experience, and skills toward achieving objectives of the intervention.

#### Government Health Officials and Workers

Government health officials have responsibility of providing healthcare to the community and they view the intervention as an opportunity to improve the delivery of services. They participated in core group meetings, listened to demands of the community, attempted changes within healthcare services and supported communities’ efforts. Changes within healthcare services included initiating outreach immunization camps and workshop on interpersonal communication. They supported community-level health promotion and education by providing health education materials and relevant details on health services offered by MCGM that was useful for preparing health communication material. They were key drivers, designers, and implementation agencies of the intervention.

#### Non-Governmental Organizations

Non-governmental organizations had their own specific health interventions, including maternal and child health, tuberculosis care, and nutrition (especially malnutrition among children), and social interventions, including access to food rations and sensitization regarding violence against women. Although they attended core group meetings and facilitated discussion between community and governmental officials, they did not play active role in planning and implementation of intervention. The NGO workers were busy with their specific interventions and could not set aside time for the present intervention.

### Context

Aspects of environment of intervention that may have a bearing on the process and relevant results are presented here. Most migrants in intervention area were inter-state migrants with mother-tongue different from the local language *Marathi*. Local politics in Mumbai views inter-state migrants as outsiders who steal employment opportunities from local job seekers that may result in their further alienation. However, migrants in this study did not report discrimination in provision of healthcare services on the basis of “status as migrant” or “place of origin.” But they reported exclusion from services due to lack of residential proof. One migrant woman reported, “*I took my neighbor for delivery to a maternity home but they asked us to go back and bring the ration card. We told the staff nurse that she did not have it as she has recently migrated (to Mumbai) and has not yet obtained one. The staff nurse said that ration card is mandatory and without it how can she register her*.” Mumbai provides jobs but not accommodation. Lower wages leave poor migrants with the only option of staying in non-notified slums where rent is lower. Government authorities cannot issue residential proof for those living in non-notified/illegal structures. This contextual factor results in their exclusion from the provision of public services, including government health services.

Local elected representatives do intervene and provide water, community toilets, roads, electricity, etc., in order to secure their vote bank. But they have little say in deciding the status of slums as notified or non-notified. One community member said, “*Nagar Sevak (local elected representative) elected from their community had asked MCGM to provide water supply to all the households in the slum cluster but no action was taken. He and other Nagar Sevak called for a protest march with participation of the community. But in spite of this there was no action by Municipal Corporation*.” Notification of slums is larger state-level political issue and local elected representatives do not have much say in it. Non-notified slums are liable for demolition by MCGM. Such actions are taken periodically that are often halted by intervention of local politicians, social activists, NGOs, and communities themselves. Nevertheless, the possibility of demolition increases the vulnerability of migrants and excludes them from civic services.

Most migrants are unaware of the location of government health facilities. Even when they are aware, their working hours coincide with those of government health facilities, thereby preventing access. Private healthcare providers with clinics in the slums are the usual source of healthcare for migrants. Women do not typically venture out as they do not know the city and rely on healthcare providers in the vicinity.

The healthcare delivery system of MCGM has been described earlier. Primary and secondary healthcare facilities have fewer resources and often result in referral of patients to tertiary care facilities. The maternity home close to the clusters was closed down due to dilapidated condition of the building toward the end of intervention. These contextual factors may affect delivery of intervention.

### Resources

Resources for intervention were largely drawn by the stakeholders themselves. External financial resources from a funding agency were used only for pre-intervention workshops, reproducing IEC material and conducting process evaluation. With respect to human resources, significant contributions came from government health officials and workers, core groups’ migrant members and CBO leaders. Core group meetings were held at houses of members of the community, community building, and offices of political parties. Expenditure on creating prototype IEC material, conducting a health camp, and providing healthcare was borne by MCGM.

Available resources were scarce. The medical officer of one urban health post said, “*We have five health workers (four auxiliary nurse midwives and one multi-purpose (male) health worker). The population catered to by (this) Health Post is 150,000 and each health worker caters to 30,000 people. Health worker population ratio norm is 1: 15,000 people but here it is much more*.” Limited resources at primary and secondary care levels result in referral of cases to tertiary facilities. For example, the maternity home had limited human resources and infrastructure and complicated pregnancies were referred to tertiary medical institutions for delivery. Scarcity of resources at MCGM limited provider-led health promotion interventions makes the role of community crucial. Potential increase in demand from the community was not paralleled with an increase in MCGM’s resources for health.

There are no funds available at the community level for development. The Municipal Corporation sets aside funds for slum development but these community clusters are non-notified and so funds are not available for them. Elected representatives from the community in Municipal Corporation have used municipal funds available to them for building community toilets, roads within community, and community meeting halls. Hence, their support for health intervention could not be sought.

### Implementation

Information, Education and Communication material on location, timing, and type of health services available at municipal health facilities was distributed by the community members in the core group and women’s self-help groups. It was displayed at prominent locations within the community as well as during festivals. Similar was the case for health education material related to common conditions of ill-health.

Cluster 3 planned for monthly community immunization camps that were conducted during the first 4 months. But demolition of slums resulted in a break in this activity. Both the community and government health officials were interested in providing this but the “torchbearers” were busy addressing basic concerns such as shelter, water, and sanitation. During the last month of process evaluation, community members were discussing with health officials the possibility to restart immunization camps. The core group had planned to have a community-level DOT provider (directly observing treatment of TB). However, it did not materialize as people who volunteered withdrew on three occasions.

The core groups had thought of exposure visits of community representatives to municipal health facilities so that they could view existing facilities, understand the administrative procedures, and interact with healthcare providers. However, this activity did not occur due to lack of planning. Also, the most important healthcare unit, the maternity home, was closed down (as mentioned earlier) and exposure to it did not occur. The training of maternity home staff was conducted as planned by MCGM’s Information, Education and Communication (IEC) wing.

One of the NGO stakeholders had suggested peer leader model to strengthen community resources. He proposed during core group meeting of cluster 2, “*People residing in one lane should identify one representative. S/he will help coordinate the intervention for that lane. Such representatives from all lanes in the cluster should attend core group meetings. They should convey health needs of community and assist in disseminating information and will have responsibilities to coordinate*.” It was proposed that the NGO will conduct health education sessions within the community for these representatives who could then communicate the messages in their respective lanes. However, representatives from lanes were neither identified, nor health education or capacity-building sessions were planned by the NGO for the core group members.

### Reach

It reached migrants living with families readily but it did not reach single male migrants. Hence, the research team had to approach single male migrants in their residential and/or workplace units. The team looked for volunteers who could take up the responsibility to spread the health education materials and messages among these industrial units, but none came forward. The health education material and messages through pamphlets were delivered to them only once during the intervention when the research team was exploring the possibility of their participation. In addition to this, messages through posters displayed in the community might have reached them.

Immunization camps were conducted in clusters 1 and 2 prior to intervention, but many recent migrants were not aware about them. The intervention attempted to make them aware of timings and location of health camps. For cluster 3, immunization camp was started as a new activity and could reach about 100 children during each camp. The reach of intervention varied across groups, being poorer among recent migrants compared to those settled. One recent migrant woman met by the research team asked, “*Do you know where and when the health camp is organized? I have young children and cannot go far to the health post; there is nobody at home to take care of them*.” When informing about it, she said, “*I do not know about this place. I am new here; I do not step out of the house*.” She was not member of any women’s group and had little interaction with others in the community.

### Barriers

Dissemination of IEC messages through interpersonal route used communication channels that existed prior to intervention. No new communication channels opened up, as stated earlier, one NGO representatives idea to have a peer leader for each lane did not find takers and single male migrants were left out from interpersonal communication. The difficulty in developing new communication channels in a short period of time was a major barrier. Another major barrier was healthcare providers’ lack of understanding of the community needs regarding the quality of services.

High mobility also affected the reach of intervention. Most migrants lived in rented accommodation. Some of them shifted to another rented accommodation during the intervention period. One group of single male migrants was interested in receiving the intervention, but did not participate as it was vacating its rented accommodation and moving out of the community cluster. Migrants visit their native place and those who had left the area temporarily did not receive some of the interventions.

Yet, another barrier was the lack of readiness to accept deficiencies within the government system. During the second pre-intervention workshop, results from a previous formative study were presented. The study found that one-fourth of Mumbai’s recent migrant women delivered at home. The government health officials were not willing to accept the findings of the study. This led to distrust between the research team and government officials. The government health officials were met in person by the research team after the workshop and possible ways to have resolution of conflict were discussed. Another workshop with detailed analysis and explanation helped address some concerns of MCGM officials. However, this lack of trust resulted in delaying intervention.

In another instance, during one core group meeting in cluster 1, the community voiced its anger against staff behavior and the nurses at maternity home who shout at and beat women in parturition. The medical officer said that it is important that labor progresses in time and many women do not understand how to deal with it. Under such circumstances, the staff needs to shout. Women clearly felt that the medical officer was insensitive to their feelings and needs, and will not be able to bring about a change in the behavior attitude of the staff. The community volunteers wondered how they could suggest women to use government services if their dignity is not kept.

### Exposure

Health education material regarding the location and timing of government health facilities was well received by migrants living with families. Single male migrants too received it but felt that this information was of no use to them. They were not interested in this knowledge. They said, “*We are not sick. Why should we know this*?”, “*We are busy with work, we do not have time to listen to all this*,” ‘*We go to a private doctor nearby and have never been seriously ill*.” They did not read the information in detail because they felt that even when ill they will not be able to take a day off from work for accessing services from MCGM. They preferred private healthcare providers who were available to them beyond working hours.

Pre-conceived notions limited exposure and effects of intervention. In one of the meetings, providers and community were discussing the use of family planning methods. One of the male member said, “*This is not permitted in our religion. The woman who undergoes a cut (family planning operation) will not get her soul ascended in heaven*.” However, female members did not agree to the idea and one woman pointed that there were other methods of family planning. Women felt that there are some individuals in the community who in the name of religion attempt to oppress women and involving such men will only hurt their interests.

Almost all migrants communicated in Hindi, but only a few could read it. This was the reason why flip charts were given to the core group members and women’s groups who could use them for disseminating information.

### Contamination

It is often difficult to separate the impact of intervention solely in improving access to healthcare as other external factors may play a role. NGOs run projects in the area of health and healthcare in the slums where these three intervention clusters were located. The NGO programs had the objective to impart health education to the community and improve its health behavior. Health education interventions may improve awareness in the community about health issues and have potential to improve access to government health services. However, NGO programs did not always have the objective to improve access to government health facilities. At times, they were also healthcare providers and, thus, competed with the government. This competition may influence the outcome of the intervention.

## Discussion

There have been global attempts of community participation for addressing health issues among migrants, predominantly for HIV prevention (30–32) to begin with but lately many other purposes (33–37). Studies that focus on community participation outside the HIV paradigm in India are rare. To the best of our knowledge, the present study is one of the first interventions to improve access to healthcare among internal migrants in India to be documented. This community-based stakeholder-led intervention was unique in various ways. First, it looks beyond disease-specific approach and attempts to improve access to promotive, preventive, and curative healthcare. Second, it is not designed or implemented by the research team but by stakeholders including community members who were the main drivers. Third, resources needed for intervention are predominantly contributed by stakeholders and funding agency covered only the cost of pre-intervention workshops and reproduction of IEC material. Fourth, the community demanded action from healthcare providers. Migrants voiced their concerns and led to changes within the government system, including communication skill workshop for its staff and provision of additional services within it.

Previous literature cautions that an intervention involving the community or multiple stakeholders is often met with “lag time”: time taken for involving stakeholders and a forging partnership among them. Several meetings are then needed to define the problems and develop strategies to address them (38). However, the present process evaluation found recruitment to be a relatively easy procedure due to pre-existing relationships among various stakeholders.

Process evaluation identifies strength of the intervention that was in participation from the core groups’ migrant members supported by government health officials. These champions/torchbearers from the community were key drivers who developed and executed the plan with little external financial and technical resources. Core group migrant members the taking front seat meant that the process was more people centered than intervention-driven and the community was a part of the health system rather than “target”. According to Whittington’s (39) classification, approach of the intervention could be termed as “systemic” where activities get defined during the process in close collaboration with the community and not pre-defined by the research team or health professionals, and provides insights into processes (38, 39).

The migrant community was not homogeneous and the participation of its members varied across its population. If we apply the wheel of participation framework (developed by South Lanarkshire Council), engagement of migrants varied from information (single male migrants and recent migrants) to consultation (settled migrants) to participation (core group members). But the intervention did not reach to stage of empowerment (40). Fienieg et al. (41) list reasons for participation from such “community champions” that were found to be true in this intervention as well (41). The reasons included desire to bring change in situation (in the present case improvement in access to government’s healthcare services), personal development and recognition within the community and to develop and maintain valued relationship with governmental agencies. Weaker participation from recent migrants and single male migrants needs evaluation. Process evaluation indicates two such reasons: context in which they live and their social networks. The contextual factors and their weaker social networks prevented them from participating in any community-level activity. The vulnerability and marginalization pose as barriers in community participation and have been documented in an African study (42).

The study also highlights successes and challenges in inclusive partnership. Partnering with government officials, local NGOs, and CBOs provided technical inputs to communities’ plans and helped shape the intervention. The government agency participated more actively as their organizational goals were aligned with the project’s goal but not of NGOs. The implementation framework did not have incentives for NGOs and other stakeholders, limiting their role as passive one. Also, NGOs could not spare their resources that were committed for various projects with stringent timelines. With respect to execution of plan, even MCGM’s role was limited to service provision as limited human resources could not be freed for community-level activities, nor did it help in skill building of community for they also lacked expertise in community mobilization and capacity building. The partners did not significantly gain in terms of strengthening themselves.

This pilot intervention was constrained by timeline of 1 year and limited external financial resources. Batten and Holdaway (43) have argued that program timelines can constrain community participation (44). In present intervention, the stakeholders did not venture beyond the main idea of improving access to governmental health facilities and basic health education regarding common health conditions. The intervention utilized existing social networks (as discussed in previous paragraph) but did not witness developing new networks nor extending existing networks or creating interactions between them. Similarly, the intervention did not include skill building of the community.

In spite of the challenges discussed earlier, the intervention was planned and implemented and the health education messages reached the migrants. The intervention proves that communities (even the marginalized communities) have resources within which they could help drive a health education intervention. This intervention led to improvement in access to healthcare for migrants. Quantitative data to support the improvement is not presented here as the main aim of this paper is to document the process of intervention rather than show the success through numbers. Dissemination of information and communication messages happened in an informal manner rather than structured health education sessions conducted by health professionals. Previous literature also indicates that health promotion can occur by improving informal social and communication processes such as sharing of information through informal conversations with community members and organizing community events (43, 45). In fact, the community-based health interventions need communication and social networking skills, apart from distinctly different professional, organizational, and policy skills (43, 46).

We examine now learning from the process evaluation for urban health policy in India, especially inclusion of migrants who are one of the most marginalized and underserved group. Present intervention brought consumers and providers of public health services together where migrants could voice their exclusion and barriers to accessing healthcare, including health system responsiveness issues like staff behavior and administrative procedures. However, these *ad hoc* interactions did not create formal mechanisms for voicing community concerns in management of health facilities. In rural India, National Rural Health Mission (NRHM) has introduced *Rogi Kalyan Samiti* (RKS which means patient welfare society or hospital society) which is a type of health facility committee concerned with managing affairs of the hospital with community participation. Currently, urban India lacks such community representation in management of health facilities which NUHM aims to bring in. Globally, there is evidence that such committees improve the quality of care but multiple factors influence their function (47).

Similarly, core groups were formed during intervention to spearhead it but no formal mechanism was established for their continuation after completion of the intervention period. In rural India, Village Health and Sanitation Committees (VHSCs) have been formed to take collective actions on issues related to health and its social determinants. For urban areas, NUHM proposes *Mahila Arogya Samiti* (Women’s Health Committee) to facilitate improved access to public and private healthcare at the community level (14). Some pilot experiments to involve urban communities in health planning have been documented which include a multi-stakeholder partnership in neighboring Navi Mumbai that led to the formation of *Vasti Arogya Samities* (Community Health Committees) with NGOs playing the role of facilitators (48). Women’s self-help groups have been formed to improve health outcomes in India and abroad (49–51). However, this process evaluation shows that women’s groups had limited reach to single male migrants and a separate model for reaching the latter is needed within NUHM. There are some positive lessons from migrant interventions for prevention of HIV/AIDS. These interventions have created peer educators from among the migrants who not only have served as a medium for health education and promotion, but have also resulted in community mobilization and formation of CBOs. Such peer educator models need to be explored for improving the access to healthcare.

Facilitation role played by the researcher also merits discussion. Could the intervention be successful without the research team? We doubt it. Although team did not design intervention or implement it, it facilitated the intervention. Its presence in the community constantly reminded other stakeholders and many core group meetings of intervention. It brought the stakeholders together. But there were no formal mechanisms of stakeholder partnership. The team’s presence made the environment conducive for partnership. Success documented in Navi Mumbai was due to facilitation in the initial phase by NGOs. Absence of facilitation can result in failure of formal mechanisms as well. Experiences in rural India show that community participation is often weak in RKSs (52–54). VHSCs also have faced challenges in community participation similar to RKS (52, 54–56). Based on the process evaluation results and review of this literature, it is felt that the presence of an external agent (probably NGO or academics) to anchor such efforts is needed in initial phases to ensure community participation and strengthen it. Health systems research shows that in order to be effective, building managerial capacity and ensuring community participation is essential for both RKSs and VHSCs (57, 58). These efforts of community engagement are only a useful first step, in longer run contextual issues of basic amenities, housing, wages, social security measures, and efforts of mainstreaming migrants are important (59).

The study had certain limitations. Process evaluation used only qualitative methods. Mixed methods would have provided more data on exposure and achievement of intervention. However, it was not feasible given the fact that the activities at community level evolved over a period of time and there was no pre-determined dose of intervention. The intervention had neither the structured sessions nor structured delivery to community. Quantifying within an evolving intervention would have been difficult. Qualitative data avoided this difficulty. After completion of the intervention period, a quantitative survey was undertaken and it included elements on exposure, reach, and use of intervention. Research team facilitated community meetings, their presence might have resulted in participants behaving differently than under non-observatory circumstances, which remains another limitation.

The study shows that inclusive partnership with migrants represented through formal community health committee, a capacity building or catalyst partner, and health department of local body holds promise for improving access to healthcare among poor migrants. Participation by various sub-groups of migrants warrants a multitude of models such as women’s committees, youth clubs, and peer leaders for single male migrants. Informal channels of communication can help deliver health promotion and education intervention in shorter time period but does not preclude need of formal community health committees. In order to improve weak participation from other stakeholders (includes NGOs), their role need to be formally defined and incentives for them to participate need to be devised and tested in field situations. NUHM can scale the intervention for the city’s poor migrants, test newer sustainable strategies for community participation and inclusive partnership, and through the same achieve its goal of ensuring healthcare for poor migrants.

## Author Contributions

BB prepared concept note on intervention design for the multi-centric National Taskforce Study. Detailed plan of intervention phase was prepared by all three. Concept note on process evaluation component of the intervention was prepared by NG. SM and NG collected data with the help of the research team. Preliminary analysis of data was done by NG. First draft of the paper was prepared by NG and revised by SM and BB. Final draft of analysis was prepared by NG and reviewed by SM and BB.

## Conflict of Interest Statement

The authors declare that the research was conducted in the absence of any commercial or financial relationships that could be construed as a potential conflict of interest.
